# Compromised quadriceps and hamstring force control, not maximal strength, is associated with gait biomechanics at 9 months following anterior cruciate ligament reconstruction

**DOI:** 10.1002/ksa.70129

**Published:** 2025-10-28

**Authors:** Beyza Tayfur, Alexa K. Johnson, Riann M. Palmieri‐Smith

**Affiliations:** ^1^ School of Kinesiology University of Michigan Ann Arbor Michigan USA; ^2^ Orthopedic Rehabilitation & Biomechanics (ORB) Laboratory University of Michigan Ann Arbor Michigan USA; ^3^ Department of Physiotherapy and Rehabilitation Kırşehir Ahi Evran University Kırşehir Türkiye; ^4^ Department of Orthopaedic Surgery Michigan Medicine Ann Arbor Michigan USA

**Keywords:** anterior cruciate ligament injuries, force control, gait analysis, isometric contraction, muscle strength

## Abstract

**Purpose:**

To understand the changes in muscle force control ability and its association to gait biomechanics following anterior cruciate ligament reconstruction (ACLR).

**Methods:**

Twenty‐five participants, 9 months following ACLR, performed isometric quadriceps (20%, 40% and 100% maximum voluntary isometric contraction [MVIC]) and hamstring (20% and 40% MVIC) contractions on an isokinetic dynamometer, tracing a target line. Torque variability using coefficient of variation, and complexity using sample entropy were calculated. Knee moments and knee flexion excursion were calculated from walking gait. Two‐way mixed model analyses of variance were used for between‐limb comparisons and Pearson's correlations to evaluate the associations between force variables and gait biomechanics.

**Results:**

Quadriceps and hamstring torque variability changed depending on the limb and the effort level. Quadriceps variability was higher in the ACLR limb only at maximum effort (*p* = 0.039, Cohen's *d* = 0.437). Hamstring variability showed no differences at 40% but lower torque variability (*p* = 0.035, Cohen's *d* = −0.448) at 20%. Muscle force complexity was consistently higher in the ACLR limb compared to the noninjured limb, regardless of effort level. Additionally, as effort increased, muscle force became less complex in both limbs.

Quadriceps variability was negatively associated with knee moments (*r* = −0.410, *p* = 0.042, better force control = higher knee extension moments). Hamstring complexity was positively associated with knee flexion excursion (*r* = 0.417, *p* = 0.038). Maximal strength was not associated with any gait biomechanics.

**Conclusions:**

Quadriceps and hamstring force control ability are altered and associated with gait biomechanics at 9 months post‐ACLR. Rehabilitation should include exercises focused on force control to potentially help restore gait biomechanics.

**Level of Evidence:**

Level IV.

**Abbreviations**
ACL: anterior cruciate ligament
ACLR: anterior cruciate ligament reconstruction
CV: coefficient of variation
MVICs: maximum voluntary isometric contractions
SampEn: sample entropy

## INTRODUCTION

Quadriceps femoris and hamstring muscle dysfunction are common following anterior cruciate ligament reconstruction (ACLR) [31]. This muscle dysfunction may include deficits in: maximum muscle strength, neural excitability of the muscle, muscle size, timing of force production (e.g., rate of torque development or electromechanical delay) and the ability to control force production (e.g., torque variability or complexity) [2, 10, 31]. The clinical importance of this dysfunction is well‐understood for quadriceps maximum strength, which is the most common measurement of neuromuscular function after ACLR, as it correlates with patient‐reported outcomes, functional performance and symmetrical lower limb biomechanics following ACLR [10, 18, 23, 34]. Other aspects of neuromuscular function, such as force control (i.e., torque variability or complexity), and their functional impacts after ACLR are less commonly investigated.

Force control is the ability to sustain a certain level of muscle force output, which can never be 100% constant but fluctuates around an average force [6]. It has been shown that ACL injury and ACLR surgery, may diminish quadriceps force control compared to the noninjured limb or healthy individuals [21]. This deficit in force control may have functional implications. For example, in healthy adults, quadriceps force control has been shown to explain more than 50% of variance in dynamic balance [17]. In the ACLR population, worse quadriceps force control is associated with worse self‐reported and clinical knee function in the long‐term (18 months postsurgery) [19]. Similarly, better quadriceps and hamstring force control is associated with better single‐leg hop test performance in ACL‐deficient participants [24].

Force control can be quantified using different methods including linear (magnitude‐based) and nonlinear (time‐based) fluctuations of force [4, 14]. Torque variability (or steadiness) is a magnitude‐based representation of force control, and is measured by calculating the amplitude of the force output fluctuations (coefficient of variation) around the average force output [6]. A lower level of torque variability (i.e., coefficient of variation) represents a smoother force output, hence better control of force. Torque complexity on the other hand, is a time‐based measurement of force control ability and quantifies variations between successive data points rather than the variation from a fixed average force output [14, 21]. Complexity represents the irregularity and adaptability of the temporal structure of the force output. As both variability and complexity measures provide different information about a person's force control ability; it has been recommended that both measures are utilised [4, 21].

Previous studies on the ACLR population showed changes in quadriceps torque variability and complexity at different time‐points following surgery [3, 8, 14, 24, 30, 32, 37]. However, none included both variability and complexity measures to understand different aspects of muscle force control. Also, these studies focused on the quadriceps femoris muscle group, but the hamstring muscles may also be affected and could have functional implications in the ACLR population [31]. Therefore, measuring both quadriceps and hamstring variability and complexity can provide valuable information about a person's functional recovery post‐ACLR.

Walking is the most performed daily functional activity. Following ACLR, deficits in walking gait biomechanics, especially in the sagittal plane, have been reported to persist for years [9, 15]. Specifically, knee flexion angles and moments remain lower in the ACLR limb in the long‐term [9, 15], and are linked to degenerative cartilage changes [38]. Although quadriceps muscle function is associated with these biomechanical changes [18], recovering maximum strength may not guarantee the recovery of these gait asymmetries [1]. Therefore, other parameters of muscle function, such as force control, have been of interest to understand the persistent gait deficits post‐ACLR [30].

The primary aim of the current study was to understand the differences in knee extensor and flexor muscle torque variability and complexity between ACLR and non‐ACLR legs at 9 months following ACLR surgery. The secondary aim was to identify the association of force control measurements to gait biomechanics to better understand the functional implications of force control ability.

## METHODS

ACLR patients who were between the ages of 14 and 45 years and underwent ACLR surgery using an autograft in a single centre were recruited at 9 months post‐ACLR for this observational, cross‐sectional study. All participants had presented to our clinic with an acute ACL rupture and had independently elected to undergo ACLR, with the specific surgical approach determined by the treating surgeon's clinical judgement. Exclusion criteria were having a revision ACLR surgery, contralateral ACLR, a multiligamentous reconstruction, or a knee dislocation at the time of injury. All participants received standard‐of‐care ACLR rehabilitation with the final phase of the rehabilitation protocol targeting strength maximisation, functional activity and an agility program that consisted of various movement activities (running, hopping, cutting, shuttle runs, etc.).

A total of 25 individuals with a unilateral ACLR surgery were included in the study. Participant demographics can be found in Table 1. Thirteen of the 25 participants underwent concomitant procedures in addition to ACL reconstruction, including lateral meniscal debridement (*n* = 6), lateral meniscal repair (*n* = 4), medial meniscal repair (*n* = 2) and medial meniscus rasping (*n* = 1). Each procedure was completed in a different patient (i.e., no patient had more than one concomitant procedure).

**Table 1:** Demographics of the study participants.

*n* = 25	
Age (years)	24.0 (8.1)
Sex	16 females, 9 males
Height (cm)	173.5 (9.8)
Mass (kg)	78.2 (16.8)
Body mass index (kg/m^2^)	25.9 (5.1)
Graft	22 patellar tendon, 3 quadriceps tendon
Time since surgery (months)	9.1 (0.4)
Knee osteoarthritis outcome score (KOOS)	
Pain	90.0 (7.7)
Symptoms	84.6 (11.2)
Activities of daily living	97.1 (3.5)
Sports	75.4 (17.2)
Quality of life	60.5 (15.3)

*Note*: All data are presented as mean (standard deviation).

### Knee extensor and flexor maximal strength

All strength measurements were recorded bilaterally starting with the non‐ACL limb, using an isokinetic dynamometer (Cybex Humac Norm, CSMI). Participants were seated in 90° of hip flexion, with the knee joint aligned with the axis of rotation of the dynamometer. The torso, waist, thigh and shank were secured with straps. Participants performed two sets of five 50% submaximal isokinetic concentric contractions, and then completed a minimum of three sets of five maximal isokinetic concentric contractions until no further increase in torque was observed, with 2 min of rest between sets. All isokinetic tests were completed concentrically at an angular velocity of 60°/s. Participants were instructed to kick out and pull back as hard as they could and verbally encouraged during the maximal tests. The highest torque value out of all repetitions was used for analysis.

Maximum voluntary isometric contractions (MVICs) were also collected for the knee extensor muscles, to enable the direct comparison of the results to previous force control studies as they used knee extensor MVICs [3, 14, 30]. The MVIC was only performed for the knee extensors and not for the knee flexors due to time constraints and to minimise participant burden. The knee was set at 60° of knee flexion on the isokinetic dynamometer (0° meaning full knee extension). Participants performed warm‐up trials as 25%, 50% and 75% of their maximal strength, a warm‐up 100% effort trial, and then two 5‐s MVICs with a minute of rest between trials. Participants were instructed to kick out as quickly and as hard as they could and were verbally encouraged throughout the muscle contraction.

The order of knee extension MVICs and the isokinetic concentric tests were randomised. These maximal tests were then followed by the force control measurements.

### Force control measurements

All force control measurements were done isometrically with the knee flexed to 60°. The maximum isokinetic knee extensor and flexor strength test results were used to set the submaximal targets for the force control measurements. The target torques were set at 20% and 40%, as the quadriceps and hamstring muscles are working at these levels during the gait cycle [27]. The reference torque magnitude was visually presented on the monitor and the participant was instructed to match the target line as accurately as possible for approximately 8 s. One practice trial was performed, followed by three repetitions with at least 30 s of rest between each muscle contraction. The tests were performed for knee extension first, and then knee flexion. The order of different target levels (20% and 40%) was randomised.

All torque data were recorded with a custom written program at 1000 Hz sampling rate (LabVIEW version 8.5; National Instruments). Out of the 8‐s contractions, the middle 5 s was used to analyse the force control. This was to ensure the torque signal represented the actual target tracking phase and not the ramping up/down phases of contraction. The onset of muscle contraction was defined as the time point at which the torque curve exceeded 7.5 Nm above baseline. We calculated the torque variability using coefficient of variation (CV) and torque complexity using sample entropy, to quantify both linear (magnitude‐based) and nonlinear (time‐based) fluctuations of torque, respectively [4, 14]. CV was calculated as follows: CV = standard deviation/mean × 100. Sample entropy was calculated as SampEn(m, r,N) = −ln(A/B), as previously described [14]. Briefly, m was the number of data points in which the distance between data points was compared to fit within and was set to 2 [25], and *r* was the similarity criterion and set to 0.2 times the standard deviation. *N* was the total window of data (middle 5 s, 5000 data points). A was the number of sequential data points (m + 1) and B was the number of sequential data points (*m*) that fall within *r*.

Knee extension MVIC (100% effort) data were also analysed for force control ability. Since this was a 5‐s contraction, the middle 3 s were analysed for CV and SampEn. All data were processed offline with a custom‐built MATLAB script (MATLAB version R2021b, MathWorks, USA).

### Gait biomechanics

Gait biomechanics were collected using a 12‐camera motion capture system sampling at 200 Hz (Qualisys AB) and two inground force plates sampling at 2000 Hz (AMTI). Individuals were outfitted with 39 reflective markers to model the trunk and lower extremities. Markers were located bilaterally on the acromion processes, anterior and posterior superior iliac spines, iliac crests, greater trochanters, thigh (anterior and lateral), medial and lateral epicondyles, tibial tubercles, shank (lateral and distal tibias), medial and lateral malleoli, calcanei, first and fifth metatarsal heads, and bases of the second metatarsals, and placed individually on the sternum, and C7 and T10 vertebrae. After marker placement, individuals conducted a static trial standing in a neutral anatomic position. Participants' self‐selected speed was then identified by having them walk between timing gates along a 15 m walkway, several times, at their most comfortable walking speed, to get their average self‐selected speed. They were then instructed to walk at this same speed, recorded to ±5% of their average, over the force plates. Information about striking the force plates was not relayed to the participant to avoid alterations in typical gait patterns. Five good trials were recorded for each limb, in which a good trial was defined as complete foot contact, for at least one foot, on the entirety of the force plate. If at least one entire foot contact did not land on the force plate the walking trial was repeated.

Kinematic and kinetic data were processed using Visual 3D (Has‐Motion) and filtered using a 4th order, zero phase‐lag lowpass Butterworth filter with a 12 Hz cutoff. Knee angles were calculated using a Cardan rotation sequence and internal knee moments were calculated using inverse dynamics and normalised to mass and height. Kinematic and kinetic data were time normalised to 100% of stance. Knee flexion excursion was calculated as the difference between the peak knee flexion angle and knee flexion at initial contact. Peak knee extension moment was identified as the maximum knee extension moment occurring during the loading response phase of stance.

### Statistical analysis

An a priori sample size calculation was performed using G*Power [7] based on a previous study exploring the correlations between force control and running biomechanics in ACLR patients [30]. A total of 25 participants was needed to achieve an effect size of 0.481, with 80% power at an alpha level of 0.05.

Between‐limb differences for maximal concentric isokinetic strength, knee moments and knee flexion excursion were analysed using paired samples *t*‐tests. Comparisons of force control measures between limbs (ACLR vs. non‐ACLR) and effort levels (20%, 40% and 100% for knee extension, and 20% and 40% for knee flexion) were made using four separate (knee extension torque variability, knee extension torque complexity, knee flexion torque variability and knee flexion torque complexity) two‐way mixed model analyses of variance. Bonferroni multiple comparison procedures were performed for significant interactions. Pearson's correlations were used to evaluate the associations between force variables (maximal strength, CV and SampEn) and gait biomechanics (knee moments and knee flexion excursion) separately for each limb. Effect sizes were estimated based on Cohen's *d* for the differences between ACLR and non‐ACLR limbs. An alpha level of 0.05 was considered statistically significant for all tests. All statistical analyses were performed with IBM SPSS Statistics Version 28.0 (SPSS Inc.).

## RESULTS

Maximum isokinetic knee extensor strength (Nm/kg) was lower in the ACLR limb when compared to the non‐ACLR limb, 2.15 ± 0.77 and 2.81 ± 0.68, respectively (*p* < 0.001, Cohen's *d* = −1.350). Maximum isokinetic knee flexor strength (Nm/kg) showed a similar pattern, with lower strength for the ACLR limb (1.72 ± 0.40) than the non‐ACLR limb (1.84 ± 0.41), (*p* = 0.008, Cohen's *d* = −0.577).

Gait biomechanics showed no differences between limbs for knee moments (ACLR: 0.36 ± 0.13, non‐ACLR: 0.40 ± 0.17, *p* > 0.05) or knee flexion excursion (ACLR: 16.31 ± 4.25, non‐ACLR: 17.50 ± 5.59, *p* > 0.05).

All torque variability and complexity results are presented in Tables 2 and 3.

### Torque variability

There was a significant limb × effort interaction for knee extension torque variability (CV) measurements (*F* = 5.028, *p* = 0.029). Post hoc analysis revealed that there was no difference between limbs at 20% or 40% effort (*p* > 0.05), but there was a significant between limb difference at 100% effort with the ACLR limb showing higher knee extension torque variability (*p* = 0.039, Cohen's *d* = 0.437) (Table 2).

**Table 2:** Knee extensor (Quadriceps) torque variability during isometric contractions at different effort levels.

	ACLR limb	Non‐ACLR limb	*p*	Effect size
Torque variability (CV) at 20%	1.82 (0.53)	2.02 (0.84)	0.222	−0.251
Torque variability (CV) at 40%	1.94 (0.58)	1.92 (0.52)	0.905	0.024
Torque variability (CV) at 100%	4.83 (2.52)	3.96 (1.82)	0.039*	0.437

Abbreviations: ACLR, anterior cruciate ligament reconstruction; CV, coefficient of variation.

* *p* < 0.05.

Knee flexion torque variability also showed a significant limb × effort interaction (*F* = 4.579, *p* = 0.043). There was no difference between limbs at 40% effort (*p* > 0.05), but the ACLR limb showed lower knee flexion torque variability at 20% effort (*p* = 0.035, Cohen's *d* = −0.448) (Table 3).

**Table 3:** Knee flexor (Hamstring) torque variability during isometric contractions at different effort levels.

	ACLR limb	Non‐ACLR limb	*p*	Effect size
Torque variability (CV) at 20%	2.00 (0.54)	2.53 (1.17)	0.035*	−0.448
Torque variability (CV) at 40%	2.49 (0.66)	2.56 (0.70)	0.674	−0.085

Abbreviations: ACLR, anterior cruciate ligament reconstruction; CV, coefficient of variation.

* *p* < 0.05.

### Torque complexity

There was no limb × effort interaction for knee extension torque complexity (SampEn) (*F* = 2.740, *p* = 0.111). However, there was a significant main effect for limb (*F* = 20.981, *p* < 0.001). The ACLR limb showed higher torque complexity compared to the non‐ACLR limb. There was also a significant main effect for effort level (*F* = 25.079, *p* < 0.001) with higher efforts resulting in lower torque complexity. Torque complexity at 100% effort level was lower than at 40% and 20%, and torque complexity at 40% was lower than 20% effort.

Knee flexion torque complexity (SampEn) showed a similar pattern with no limb × effort interaction (*F* = 3.172, *p* = 0.088) but significant main effects of both effort level (*F* = 34.149, *p* < 0.001) and limbs (*F* = 5.715, *p* = 0.025). The ACLR limb showed higher torque complexity compared to the non‐ACLR limb. Higher effort level (40%) resulted in lower complexity compared to the lower effort level (20%) regardless of limb. All torque complexity results are presented in Table 4.

**Table 4:** Knee extensor (Quadriceps) and flexor (Hamstring) torque complexity during isometric contractions at different effort levels.

	ACLR limb	Non‐ACLR limb
Knee extensor torque complexity (SampEn)at 20%	0.17 (0.11)	0.11 (0.04)
Knee extensor torque complexity (SampEn) at 40%	0.11 (0.04)	0.09 (0.03)
Knee extensor torque complexity (SampEn) at 100%	0.06 (0.04)	0.04 (0.02)
Knee flexor torque complexity (SampEn)at 20%	2.00 (0.54)	2.53 (1.17)
Knee flexor torque complexity (SampEn) at 40%	2.49 (0.66)	2.56 (0.70)

Abbreviations: ACLR, anterior cruciate ligament reconstruction; SampEn, Sample entropy.

### Associations between Torque variability, complexity and gait biomechanics

The ACLR limb showed a significant negative correlation between knee extension moment and knee extension torque variability at 40% effort (*r* = −0.410, *p* = 0.042), meaning that people with better knee extensor force control exhibited higher knee extension moments during gait. A positive correlation was also found between the knee flexion excursion and knee flexion complexity at 40% effort (*r* = 0.417, *p* = 0.038) in the ACLR limb. This indicates that having higher knee flexion force complexity was associated with more knee flexion excursion during gait. Maximal strength of knee extensors or flexors did not show any associations with knee moments or knee flexion excursion for the ACLR limb (*p* > 0.05).

For the non‐ACLR limb, there were no correlations between any of the force measures (maximal strength, variability or complexity) and gait biomechanics (knee extension moment or knee flexion excursion) (all *p* > 0.05).

## DISCUSSION

The main aim of this study was to compare knee extensor and flexor muscle torque variability and complexity between ACLR and non‐ACLR limbs at 9 months following ACLR surgery. Knee extensor torque variability was higher in the ACLR limb only at maximum effort, showing that variability measurements may require maximal effort to reveal force control deficits. For the knee flexors, variability was lower in the ACLR limb at low effort (20% MVIC), indicating better force control, with no differences at 40%. Differently, torque complexity was higher in the ACLR limb and at lower effort levels for both muscles. Complexity measures revealed between‐limb differences at all effort levels, making them more sensitive than variability for detecting altered force control.

The secondary aim was to examine how force control relates to gait biomechanics. Knee extension torque variability was negatively associated with knee moments, meaning smaller force variation was associated with higher knee extension moments. Knee flexion torque complexity was positively associated with knee flexion excursion, meaning more complex knee flexor contractions were associated with more knee flexion excursion during gait. Maximal strength showed no association with gait variables, suggesting that force control may be more relevant than strength in explaining gait alterations after ACLR.

We assessed force control using torque variability (linear, magnitude‐based) and complexity (nonlinear, time‐based) at different effort levels (20%, 40% and 100% MVIC) [4, 14]. Knee extension torque variability was only different at 100% MVIC, with ACLR limb showing higher variability, meaning worse force control. This finding aligns with prior studies using maximal effort tasks, which also reported greater variability in ACLR or ACL‐deficient limbs compared to the contralateral limb or healthy controls [5, 8, 24, 30]. At submaximal levels, however, we did not find any between‐limb differences for quadriceps torque variability. Hollmann et al. [11] Skurvydas et al. [29] and Ward et al. [37] using similar submaximal contraction levels also showed no between‐limb differences, supporting our findings. Notably, Ward et al. [37] observed bilateral deficits compared to healthy controls, suggesting a possible global effect of injury. Although we did not include a healthy control group, the lack of asymmetry at submaximal efforts may reflect either bilateral neuromuscular effects or the limited sensitivity of variability measures at lower intensities. Overall, these findings suggest that torque variability may only detect quadriceps force control deficits at maximal effort. Therefore, future studies using variability as an outcome should consider including high‐effort tasks to better capture neuromuscular impairments following ACLR.

Knee extensor and flexor torque complexity was higher in the ACLR limb. Torque variability and complexity represents different aspects of muscle function; therefore, can be interpreted differently. Higher variability reflects difficulty in producing smooth, consistent force, whereas higher complexity may indicate the neuromuscular system's capacity to adaptively regulate force across time. Complexity measures may be more sensitive to subtle neuromuscular changes, even when variability appears unchanged [11, 35]. This difference likely stems from the underlying computations: variability compares each value to an average, while complexity evaluates the pattern and structure of fluctuations over time. By incorporating temporal dynamics, complexity measures may better reflect changes in motor unit recruitment and muscle fibre contraction. In our study, complexity was identified between‐limb differences even at low effort levels, where variability did not, suggesting that complexity may be a more sensitive indicator of altered neuromuscular function following ACLR.

Increased complexity could be interpreted as better function, reflecting better adaptability of muscle force. Research on healthy individuals show that fatigue decreases torque complexity, limiting the adaptability of the force output [22]. Similarly, while tracing a constant‐force level task, older adults produce a less complex force output when compared to healthy younger people, indicating reduced adaptability [35]. However, research in the ACLR population is limited and inconsistent. Previous studies by Johnson et al. [14] and Bodkin et al. [3] reported higher complexity in the ACLR limbs at maximal effort, and Hollman et al. [11] at submaximal effort levels. Considering the ongoing functional problems in the ACLR populations, higher complexity, in this context, may be considered a deficit rather than better adaptability. Skurvydas, differently, found lower complexity in the ACL deficient limbs at submaximal effort levels [29]. While these differences may be a result of the calculation method of complexity, it is clear that muscle complexity is affected in the ACL limb and is different than the non‐ACL limb. These may suggest an optimal range of adaptability for optimal function, and lower or higher levels of complexity outside of this range may represent disability [28].

Hamstring force control in the ACL‐injured population has been investigated by only a few studies, with no differences in variability or complexity at submaximal effort levels, but greater variability in ACL‐deficient limbs during maximal contractions [11, 24, 29]. In our study, hamstring force control was assessed only at 20% and 40% of MVIC, limiting direct comparisons with studies like Pua et al., who observed higher variability at maximal effort [24].

However, we found no differences in torque variability at submaximal contractions but at the maximal contractions for quadriceps muscle. Since we did not measure maximal hamstring contractions, we do not know if we would have found a similar pattern for the hamstring torque variability. Pua's findings of higher torque variability in the ACL deficient knees at maximal effort suggest that it might be possible, since variability measurements are likely affected by effort level. Increased task demand at maximal contractions lead to a more irregular motor unit discharge rate [35], hence, worse variability may be expected. Interestingly, our finding of lower variability, hence better force control, of hamstrings in the ACLR limb at 20% effort is contrary to what might be expected. Hamstring muscle function, as measured by maximal strength may be negatively affected following ACLR regardless of the graft type, specifically in the short term [31] we similarly found lower hamstring strength in the ACLR limb. This suggests that our participants had not achieved recovery of maximal capacity of muscle force; however, they may use their ACLR‐limb flexor muscles better than the non‐ACLR limbs at submaximal levels to trace a constant force. Our participants have been through a comprehensive rehabilitation protocol following surgery and performed controlled movements focusing on constant force output at submaximal effort exercises in the ACLR limbs as part of their standard of care. Therefore, we may speculate that performing functional tasks and hamstring focused exercises throughout their rehabilitation may have helped with hamstring submaximal force control ability.

The changes we identified in quadriceps and hamstring force control ability might be affected by the time postsurgery. The participants in this study were 9 months post‐ACLR, while Hollman, Skurvydas and Pua included presurgery, ACL deficient participants in their studies [11, 24, 29]. Johnson et al. [14] reported no differences in quadriceps torque complexity presurgery, but higher complexity in the ACLR‐limb at 5‐ and 9‐month postsurgery. Therefore, ACLR surgery likely affects force control ability of the knee muscles. This might explain why Hollman or Skurvydas found no between‐limb differences in hamstring force control at submaximal contractions [11, 29], while we found lower variability in the ACLR limb. Therefore, recovery trajectories of muscle force control should be considered when designing targeted intervention strategies. As such, focusing on force control following surgery might be an important contributor to neuromuscular recovery.

It is important to acknowledge that all participants in this study received grafts involving the quadriceps musculature, including patellar tendon (88%) and quadriceps tendon (12%) autografts. This limits the generalisability of our findings to ACLR patients who receive other graft types, particularly hamstring tendon autografts. Previous research has demonstrated that the recovery trajectories of quadriceps and hamstring muscle function differ depending on graft type, with hamstring tendon harvest specifically affecting knee flexor muscles [13, 26]. As such, caution should be taken when extrapolating these findings to the broader ACLR population, especially those with hamstring‐based reconstructions. Future studies may compare quadriceps force control among patients with quadriceps tendon, patellar tendon and hamstring tendon autografts to better understand how graft selection influences neuromuscular recovery and to guide more tailored rehabilitation approaches [36].

In addition to including only participants with quadriceps‐related grafts, another limitation of this study is the absence of a healthy control group, which restricts our ability to determine whether neuromuscular impairments were also present in the contralateral, noninjured limb—an important consideration when interpreting limb asymmetries and potential bilateral neuromuscular deficits.

Furthermore, this was a cross‐sectional study conducted at a single time point (9 months post‐ACLR), which limits our ability to assess the progression or recovery of neuromuscular function over time. While 9 months is a clinically relevant stage in ACLR rehabilitation, our findings are limited to this specific time point. Future studies with longer‐term follow‐up are needed to better understand the trajectory and persistence of these force control impairments.

The functional consequences of altered force control are important to understand so that we can better appreciate the clinical implications of these lab‐based tests. Using maximal force control tests, Pua found that hamstring variability was associated with hop velocity, and quadriceps variability with hop distance and velocity [24]. Spencer reported higher quadriceps variability leading to greater knee flexion excursion during running [30]. Maximal strength (e.g., Peak torque) measurements represent system capacity. However, most activities of daily life require submaximal muscle activation. Therefore, submaximal measurements are likely more relevant to understanding the neuromuscular impairments people experience following ACLR. We found that quadriceps variability and hamstring complexity during submaximal contractions were associated with gait biomechanics. Better quadriceps control (e.g., low variability) was associated with higher knee extension moments. Knee loading in the sagittal plane has been shown to be lower than the contralateral limb for up to 6 years following ACLR [15]. Therefore, our finding highlights a potential solution for this long‐term deficit. Focusing on improving quadriceps force control during rehabilitation may help resolve this biomechanical problem. Similarly, we found that higher hamstring force complexity is associated with more knee flexion excursion during gait. This again shows better adaptability of hamstring muscle forces may lead to higher knee flexion angles, which remains low in the ACLR population in the long‐term [9]. Resolving these biomechanical deficits may lead to better function and patient‐reported outcomes [20]. Thus, focusing on improving force control and adaptability during rehabilitation may lead to better outcomes following ACLR.

Evidence supports the effectiveness of several rehabilitation strategies in improving muscle force control following ACLR. Controlled low‐load contractions have been shown to enhance neuromuscular precision and fine motor control, particularly during submaximal tasks [16]. Strength training that targets both maximal and explosive force production can further improve the magnitude and consistency of muscle output, contributing to better overall control [12]. Additionally, heavy‐load steadiness training—where individuals are required to match knee joint movement to a constant‐velocity template—may directly enhance force steadiness and dynamic joint control [33]. Integrating these approaches into rehabilitation programs may improve gait biomechanics and functional recovery by addressing force control deficits that persist after surgery.

## CONCLUSION

Our findings revealed reduced control of knee extensor and flexor muscle contractions that were associated with alterations in gait biomechanics at 9 months post‐ACLR. Quadriceps and hamstring variability and complexity are affected, with complexity being more sensitive to detect changes than variability measures. Better quadriceps control and higher hamstring adaptability are also associated with higher knee moments and more flexion excursion during gait, respectively. Rehabilitation protocols should include exercises focusing on muscle force control, which may ultimately help recover the persistent biomechanical alterations following ACLR.

## AUTHOR CONTRIBUTIONS

**Beyza Tayfur**: Idea; design; data collection; analysis; write up. **Alexa K. Johnson**: Idea; design; analysis; write up (revisions). **Riann M. Palmieri‐Smith**: Idea; design; analysis; write up (revisions); funding.

## CONFLICT OF INTEREST STATEMENT

The authors declare no conflicts of interest.

## ETHICS STATEMENT

University of Michigan Medical School Institutional Review Board, HUM00144992. Informed consent was obtained from all participants prior to any data collections. The study was approved by the University's institutional review board and informed consent was obtained from all participants prior to any data collections.

## Data Availability

The data that support the findings of this study are available from the corresponding author upon reasonable request.
